# The capacity of informal caregivers in the rehabilitation of older people after a stroke

**DOI:** 10.17533/udea.iee.v39n2e03

**Published:** 2021-06-15

**Authors:** Mariane Lurdes Predebon, Fernanda Laís Fengler Dal Pizzol, Naiana Oliveira dos Santos, Carla Cristiane Becker Kottwitz Bierhals, Idiane Rosset, Lisiane Manganelli Girardi Paskulin

**Affiliations:** 1 Nurse, Master´s degree. Unimed Porto Alegre, Porto Alegre, RS, Brasil. Email: predebon11@gmail.com. Corresponding Author Unimed Porto Alegre Porto AlegreRS Brasil predebon11@gmail.com; 2 Nurse, Ph.D. student. University of Alberta, Edmonton, AB, Canada. E-mail: fenglerfernanda@gmail.com University of Alberta University of Alberta EdmontonAB Canada fenglerfernanda@gmail.com; 3 Nurse, Ph.D. Assistant Professor, Universidade Franciscana, Santa Maria, RS, Brazil. Email: naiaoliveira07@gmail.com Universidade Franciscana Santa MariaRS Brazil naiaoliveira07@gmail.com; 4 Nurse, Ph.D. Universidade Federal do Rio Grande do Sul -UFRGS-, Porto Alegre, RS, Brazil. Email: carlakot@yahoo.com.br Universidade Federal do Rio Grande do Sul Universidade Federal do Rio Grande do Sul -UFRGS- Porto AlegreRS Brazil carlakot@yahoo.com.br; 5 Nurse, Ph.D. Assistant Professor at UFRGS, Porto Alegre, RS, Brasil. Email: idiane.rosset@ufrgs.br Universidade Federal do Rio Grande do Sul UFRGS Porto AlegreRS Brazil idiane.rosset@ufrgs.br; 6 Nurse, Ph.D. Associate Professor at UFRGS, Porto Alegre, RS, Brasil. Email: paskulin@ufrgs.br Universidade Federal do Rio Grande do Sul UFRGS Porto AlegreRS Brazil paskulin@ufrgs.br

**Keywords:** caregivers, aged, stroke, nursing, cuidadores, anciano, accidente cerebrovascular, enfermeros, cuidadores, idoso, acidente vascular cerebral, enfermagem.

## Abstract

**Objective.:**

To characterize informal caregivers of dependent older people after a stroke related to aspects of care, and to describe the activities performed and the difficulties faced by these caregivers.

**Methods.:**

Cross-sectional, descriptive study, held in southern Brazil with 190 informal caregivers of older adults after stroke. The sociodemographic data instrument and the Capacity Scale for Informal Caregivers of Elderly Stroke Patients (ECCIID-AVC), adapted and validated for use in Brazil by Dal Pizzol *et al.*, were used.

**Results.:**

Most caregivers were women (82.6%) or children (56.3%), had average schooling of 9.6 years, and the majority (68.3%) provided care for people with moderate to severe disability. The main activities carried out included: providing materials and/or support for eating (99%), dressing (98.4%), and administering medications (96.2%). Caregivers had the most difficulty with transferring and positioning activities.

**Conclusion.:**

Most caregivers have adequate capacity to provide essential care to the dependent older adult after a stroke. However, a significant portion had difficulty in the activities of transferring and positioning the older person due to the lack of guidance regarding the posture to carry out these activities. The assessment of nurses regarding the activities performed and the difficulties faced by caregivers is an important strategy to identify problems and effectively attend to the needs of these individuals at all levels of health care.

## Resumen

**Objetivo.:**

Caracterizar a los cuidadores informales de personas mayores dependientes tras un ictus en cuanto a los aspectos relacionados con los cuidados y describir las actividades realizadas y las dificultades a las que se enfrentan.

**Métodos.:**

Estudio transversal y descriptivo realizado en el sur de Brasil con 190 cuidadores informales de ancianos tras sufrir un accidente cerebrovascular (ACV). Se utilizó un formulario de datos sociodemográficos y la Escala de Capacidades del Cuidador Informal de Ancianos Dependientes por Accidente Cerebrovascular (ECCIID-AVC) adaptada y validada por Dal Pizzol *et al.*

**Resultados.:**

La mayoría de los cuidadores eran mujeres (82.6%), hijos (56.3%), con 9.6 años de educación en promedio y el 68.4% cuidaba de personas con discapacidades entre moderadas a graves. Las principales actividades realizadas fueron proporcionar materiales y/o apoyo para la alimentación (99%) y el vestido (98.4%), así como ocuparse de la medicación (96.2%). El traslado y la posición de los ancianos fueron las actividades en las que los cuidadores presentaron más dificultad debido a la falta de orientación sobre la postura adecuada que debían tener para realizar estas actividades.

**Conclusión.:**

La mayoría de los cuidadores tienen una capacidad adecuada para brindar los cuidados esenciales a los ancianos dependientes después de un ictus, sin embargo, una parte importante de ellos enfrentan dificultades que deben ser identificadas por enfermería con el fin de intervenir las necesidades educativas de estos sujetos en todos los niveles de la atención sanitaria.

## Resumo

**Objetivo.:**

Caracterizar cuidadores informais de idosos dependentes após Acidente Vascular Cerebral (AVC) quanto a aspectos relacionados ao cuidado e descrever as atividades realizadas e as dificuldades enfrentadas por esses cuidadores.

**Método.:**

Estudo transversal, descritivo, realizado no Sul do Brasil com 190 cuidadores informais de idosos após AVC. Utilizaram-se formulário de dados sociodemográficos e Escala de Capacidades do Cuidador Informal de Idosos Dependentes por Acidente Vascular Cerebral (ECCIID-AVC) adaptada e validada por Dal Pizzol *et al*.

**Resultados.:**

Os cuidadores, em sua maioria, eram do sexo feminino (82.6%), filhos (56.3%), com escolaridade média de 9.6 anos e 68.4% cuidavam de pessoas com incapacidade moderada a severa. As principais atividades realizadas foram fornecer materiais e/ou apoio para alimentar-se (99%) e vestir-se (98.4%), bem como cuidar das medicações (96.2%). Transferência e posicionamento foram as atividades que os cuidadores apresentaram maior dificuldade.

**Conclusão.:**

A maioria dos cuidadores possui adequada capacidade para realizar os cuidados essenciais ao idoso dependente após AVC, no entanto uma parte significativa apresentava dificuldade nas atividades de transferência e posicionamento do idoso, devido à falta de orientação quanto à postura adequada para realizar essas atividades. A avaliação dos enfermeiros quanto às atividades realizadas e às dificuldades enfrentadas pelos cuidadores é uma importante estratégia para identificar problemas e intervir nas necessidades desses sujeitos em todos os níveis de atenção à saúde.

## Introduction

Stroke is a neurological syndrome with a high incidence in the older population. In Brazil, approximately 153 thousand people between January and December 2020 were hospitalized due to stroke, in which approximately 110 thousand (72%) were older people, and around 18 thousand older adults (16.4%) died.(1) These data highlights the need for special attention to the older population with stroke. Usually, older people with stroke remain with some functional disability, needing help to carry out activities of daily living, which results in challenges for the family, as the demand for care does not cease at the time of hospital discharge. Thus, there is a need to choose a person to assume the role of caregiver.(2) In Brazil, most caregivers of older people after a stroke are informal.(3) Informal caregivers are those who perform non-professional care, without employment or remuneration, and may be a family member, friend, neighbor, or other people in the community.(4)

The informal caregiver plays a fundamental role in rehabilitation of dependent patients after a stroke at home, but he/she needs support to provide qualified care. (2) Although some studies show the characteristics of these caregivers and the care provided, they do not specify the activities of care and the difficulties faced due to the demands of care.(5,6) In Brazil, the government has reduced responsibilities to supporting the older population, attributing to the family the responsibility for most of the home care to dependent people.(7) Also in Brazil, informal care has not yet entered into the public policies,(8) making the family to provide care of the older adult without the necessary information and resources. However, at an international level, the support networks for the older people and their informal caregivers seem to be more strengthened, mitigating the negative repercussions of dedication to caring for dependent older people after stroke. In Canada, for example, the older patients and their caregivers have numerous public policies that aim to provide financial support to informal caregivers, adaptations in the homes of the older person, and information programs for caregivers and the older patient, guiding them on how to provide care and take care of their physical and mental health.(9)

In this context, it is the nurses’ responsibilities to use their skills as an educator to assist the family in the continuity of patient care after hospital discharge.(2) Nurses who work in the most varied points of care in the health care network, such as hospitals, Primary Health Care, and Home Care, play a fundamental role in guiding and supporting family members of the older patients through pre-discharge preparation and home visits after discharge. Thus, getting to know the informal caregivers of older people after a stroke and the characteristics of this work is essential for planning health care, improving educational strategies, and being able to direct effective public policies, which take into account their potential and limitations.

Given the above, the objective of this study was to characterize informal caregivers of dependent older people after a stroke related to aspects of care. Furthermore, it aims to describe the activities performed and the difficulties faced by these caregivers.

## Methods

This is a cross-sectional, descriptive study with informal caregivers of older people after stroke who are being followed up in one of the outpatient clinics of two public hospitals in the city of Porto Alegre, Rio Grande do Sul, Brazil, or in the Home Care Service in the same region. These services are part of the *Rede Brasil AVC* and receive patients through a health care network linked to the Mobile Emergency Care Service. The sample was derived from a larger research project,(10) and calculated from the recommendations of Beaton(11-13) and Hair(14) that foresee the inclusion of at least 30 individuals in the pre-test and the use of at least five times more individuals per item than the number of items to be analyzed. Thus, the study sample was characterized by 30 pre-test individuals + 160 individuals (scale with 32 items with five observations each), totaling a non-probabilistic sample of 190 informal caregivers of the older patients (60 years old or more) after a stroke.

We included caregivers (aged at least 18 years old) of older people with a medical diagnosis of stroke and informal caregivers who had taken care of the family member after hospital discharge for at least 15 days and at most 12 months. This temporal delimitation is because we consider it necessary for the caregiver to have the experience of providing care for the older adult after a stroke so that we could identify the activities performed and the difficulties faced by them. In this sense, we considered that, after 12 months, the caregiver has already acquired the necessary skills in the care process, without presenting the initial difficulties. Also, we selected older adults with a minimum score of two and a maximum of five at the time of discharge from the Modified Rankin Scale (mRankin)(15). MRankin is a scale that allows the functional evaluation of stroke patients, applied during the patient's hospitalization and discharge. The mRankin score ranges from zero to six (0-asymptomatic; 1-symptoms without disability; 2-mild disability; 3-moderate disability; 4-moderately severe disability; 5-severe disability; 6-death). This scale was not validated for Brazil, but it is widely used in clinical practice and research. (15) We excluded the caregivers who did not answer the calls after three attempts on different days and shifts, and caregivers of older adults who lived in long-term facilities.

We collected data from May to December 2017 in person, before or after the outpatient health consultation, or by telephone. We had 52 face-to-face data collections and 138 by telephone contact, according to the caregiver's availability. The care activities were not observed, we considered the caregivers’ report on how they performed the activities, as data were collected in an outpatient area or by telephone contact. For that, we used a questionnaire of sociodemographic data of the caregivers, with the following variables: age, gender, education, marital status, and occupation. We also asked questions to informal caregivers such as degree of kinship with the older person, live with the older person, time spent in the activity of caregiver, age of the older person, presence of another person to assist in the provision of care, time spent per week to provide care (including, in addition to physical care, care management, and emotional, financial and instrumental support for the older person), weekly time spent by others in providing care for the older adult, the income of the older person, expense taken from the caregiver's income for expenses with the older person, and receiving or not receiving financial aid. Also, some data were extracted from the computerized system of the two institutions under studies, such as: length of hospital stay for the older patient, the time between hospital discharge and the interview, mRankin score, and type of stroke. The activities for which the caregiver helped the older person were extracted from the sum of the items “demonstrates” (2) and “demonstrates completely” (3) of the Capacity Scale for Informal Caregivers of Elderly Stroke Patients (*Escala de Capacidades do Cuidador Informal de Idosos Dependentes por AVC - ECCIID-AVC*).(10) The difficulties faced by caregivers were obtained from the sum of the items “does not demonstrate” (0) and “demonstrates partially” (1) of the same scale. These last two items correspond to the difficulties faced in providing care to dependent older people due to a stroke because the caregivers do not demonstrate the ability to perform some care activity or because they proved to be partially capable, needing help from other people. The items that compose the *ECCIID-AVC* are items related to essential to care performed by informal caregivers of the older adult after a stroke, such as: food, medications, skin care, personal hygiene, bathing, eliminations, clothing, transfer, and positioning.(10)

The *ECCIID-AVC* was adapted and validated for use with informal caregivers of older adults after a stroke in Brazil, presenting a satisfactory test-retest reliability (intraclass correlation coefficient = 0.94; 95% confidence interval = 0.91-0.96) and an excellent internal consistency reliability (Cronbach’s alpha = 0.914).(10) This scale evaluates the different capacities that informal caregivers have or need to improve in providing care for dependent older people after a stroke. This is the first specific existing scale to assess the ability of informal caregivers of dependent older people after a stroke.(10,16) The *ECCIID-AVC* has 29 items that evaluate several factors related to provide care, with the following answer options: “NA - not applicable”; "0 - Does not demonstrate": Not performing the activity; "1 - Demonstrates partially": needs help to perform the activity; "2 - Demonstrates": can perform the activity with encouragement and/or supervision; “3 - Demonstrates completely”: performs the activity correctly and autonomously.(10) The answer option “NA - not applicable” is used in cases in which the caregiver does not perform the activity because the older person does not need that care, such as care with a nasoenteric tube for older people with an oral diet. In this study, the percentage of continuous variables was calculated on the number of caregivers who needed to perform the activity, discounting the cases of NA for each item. The total score of the scale varies from 0 to 87 points and the calculation is done proportionally, that is, the options marked as NA - not applicable are considered.(10) Thus, a caregiver who does not perform the activity for not having to do it (independent older person) will not be compared to those who do not, for not knowing how to do it. The higher the score, the more qualified the caregiver. The total score was calculated by averaging the scores of the responses, ranging from 0 to 3. The scale has no cut-off point.

The *ECCIID-AVC* is a scale intended to be applied by nurses or health professionals, with the professional assigning the score based on the observation of the performance of the care activities or through the report of the caregivers. In this study, the *ECCIID-AVC* was applied by a properly trained team, who questioned each item on the scale to the caregivers, assigning the score according to the caregiver's report. The interviewers used a guide for the application of the *ECCIID-AVC*, in which there were questions directed to each item of the scale so that the interviewer had criteria to assess, based on the caregiver's report, if he/she performed the activity correctly and autonomously. For example, in the item related to the preparation of food, the question was directed to identify whether or not the caregiver prepared the meal according to the prescribed or guided diet.(10,16)

For statistical analysis, we used the software Statistical Package for the Social Sciences (SPSS) version 21.0. The continuous variables were described as mean and standard deviation, or median and interquartile range. The categorical variables were presented as frequencies and proportions. The study was approved by the Research Ethics Committee under numbers 160580 and 17152. All respondents agreed to participate in the research by signing the Informed Consent Form or consent by telephone contact.

## Results

There was a predominance of female caregivers (82.6%) with a mean age of 50 ± 13 years old (Table 1). More than half of the sample (71.6%) was married or lived with partners, 38.4% were employed and had an average of 9.6 years of study. As for the degree of kinship between the caregiver and the older adult, most of them were children (56.3%), who lived with the older person (73.7%). The average age of the older adult was 72.8±10.2 years old.

The median hours per week of care provided to the older person reported by the caregivers was 142.5. This weekly care period involves physical care for the older person, care management, and emotional, instrumental, and financial support. Within this weekly workload, 78.9% of caregivers received help from other family members and friends, with a median of 70 hours per week. Among the caregivers, 101 (53.1%) had some expense taken from their income, and 97 (51%) received financial assistance from someone else. Most of the older adults, 166 (87.4%), had their income, with an average of 1.3 minimum wages.

The median time of care provided by caregivers to the older person was 5 months. Also, the median length of stay for the older person was 11 days and the median time between hospital discharge and the interview was 136 days. According to the mRankin scale applied at the time of hospital discharge of the older person, 130 (68.4%) caregivers cared for older people with moderate to severe disability, and most of them (172.5%) were cases of older people after an ischemic stroke.

**Table 1:** Sociodemographic characteristics and information related to the care provided by 190 informal caregivers of the older adults after a stroke

Sociodemographic characteristics	Descriptive statistics
Female; n (%)	157 (82.6)
Age (years old); mean±SD	50±13
Marital Status; n (%)	
Single/never married	36 (18.9)
Married/living with a partner	136 (71.6)
Divorced/Separated	15 (7.9)
Widow	3 (1.6)
Education level (years); mean±SD	9,6±3,9
Occupation; n (%)	
Employed	73 (38.4)
Unemployed	36 (18.9)
Housewife	36 (18.9)
Retired	43 (22.6)
Student	1 (0.5)
Not informed	1 (0.5)
Information related to the care provided	
Degree of kinship; n (%)	
Children	107 (56.3)
Partner	54 (28.4)
Grandchildren	7 (3.7)
Siblings	5 (2.6)
Other	17 (8.9)
Hours/week dedicated to caring for the older person; median (Q1-Q3)	142.5 (60-168)
Receive assistance from other people to assist in the provision of care; median (Q1-Q3)	150 (78.9)
Hours/week when receiving help from other people; median (Q1-Q3)	70 (28-168)
Time in which the older person is cared for (months); median (Q1-Q3)	5 (2-10)
Length of hospitalization of the older person (days); median (Q1-Q3)	11 (7-17)
mRankin; n (%)	
Slight disability (2)	60 (31.6)
Moderate disability (3)	41 (21.6)
Moderately severe disability (4)	51 (26.8)
Severe disability (5)	38 (20)
Stroke type; n (%)	
Ischemic	172 (90.5)
Hemorrhagic	15 (7.9)
Transient ischemic attack	3 (1.6)

Regarding care activities (Table 2), more than 89% of caregivers performed all the necessary activities related to oral preparation, control, and diet intake. Of the 190 caregivers, only 15 (7.9%) provided care for the older person who received a diet through an enteral tube. Of these 15 caregivers, two caregivers did not perform the introduction of water if the tube became obstructed during the administration of the diet and medication, but all performed the washing of the tube with water after administration of the diet and medication.

The main activities performed by the caregivers were to provide materials and/or support necessary to facilitate feeding (99%), to dressing (98.4%), and administration of medication (96.2%). The skin care activity of the older adult was developed by 66.3% of the caregivers and 63.1% stimulated the movement of the affected body member of the older adult to get the food and utensils. Regarding care with the transfer of the older person, 17.4% of caregivers did not need to perform it, as the older person was independent in this activity. Of the 157 caregivers who needed to transfer the older adult, 53.5% had difficulty and reported making the transfer of the older person without paying attention to their own posture. This difficulty was also present in the activity of positioning the older adult because, of the 134 caregivers who needed to position them, 43.3% also reported not paying attention to their own posture. The total average of the response options of the caregivers in the *ECCIID-AVC* was 2.56±0.42.

**Table 2:** ECCIID-AVC: Care activities and difficulties faced by 190 informal caregivers of dependent older people after stroke

ECCIID-AVC items	NA* *n* (%)	**Need to perform the activity *n* (%)**	**Does not demonstrate (0) / Demonstrates partially (1) *n* (%)** ^†^	Demonstrates (2)/ Demonstrates completely (3) *n* (%)^†^
1- Prepare meals in accord with the prescribed or guided diet plan.	10 (5.3)	180 (94.7)	19 (10.6)	161 (89.4)
2- Prepare meals adequately.	6 (3.2)	184 (96.8)	12 (6.5)	172 (93.5)
3- Place food and utensil on the side on which the elderly person shows greater dependence to stimulate the affected member.	33 (17.4)	157 (82.6)	58 (36.9)	99 (63.1)
4- Provide support and/or materials necessary to facilitate feeding.	3 (1.6)	187 (98.4)	2 (1)	185 (99)
5- Control food ingestion.	1 (0.5)	189 (99.5)	8 (4.2)	181 (95.8)
6- Monitor swallowing.	5 (2.6)	185 (97.4)	8 (4.3)	177 (95.7)
7- Aid in the administration of medication as per the medical prescription.	6 (3.2)	184 (96.8)	7 (3.8)	177 (96.2)
8- Flush the tube with water if it becomes obstructed during the administration of diet and medication.	175(92.1)	15 (7.9)	2 (13.3)	13 (86.7)
9- Flush the tube with water after administering the diet and medication.	175(92.1)	15 (7.9)	0	15 (100)
10- Hydrate the skin.	9 (4.7)	181 (95.3)	60 (33.1)	121 (66.9)
11- Prepare hygiene material.	11 (5.8)	179 (94.2)	9 (5)	170 (95)
12- Provide support and/or materials necessary to facilitate personal hygiene.	11 (5.8)	179 (94.2)	7 (3.9)	172 (96.1)
13- Assist while bathing.	21 (11.1)	169 (88.9)	27 (16)	142 (84)
14- Assist with oral hygiene.	41 (21.6)	149 (78.4)	13 (8.7)	136 (91.3)
15- Maintain a neat appearance.	7 (3.7)	183(96.3)	4 (2.2)	179 (97.8)
16- Ensure privacy while using the toilet, when changing diapers and bathing.	6 (3.2)	184 (96.8)	13 (7)	171 (93)
17- Provide support and/or materials necessary to facilitate urinary and intestinal toilet routines.	21 (11.1)	169 (88.9)	10 (5.9)	159 (94.1)
18- Assist in toilet wiping and changing diapers.	44 (23.2)	146 (76.8)	13 (8.9)	133 (91.9)
19- Provide support and/or materials necessary to facilitate dressing.	5 (2.6)	185 (97.4)	3 (1.6)	182 (98.4)
20- Assist the person while dressing.	14 (7.4)	176 (92.6)	8 (4.5)	168 (95.6)
21- Assess the elderly person’s capacity to transfer themselves from one place to another.	12 (6.3)	178 (93.7)	5 (2.8)	173 (97.2)
22- Explain to the elderly person about the right way to transfer themselves from one place to another.	21 (11.1)	169 (88.9)	8 (4.7)	161 (95.3)
23- Provide support and/or materials necessary for the elderly person to transfer themselves from one place to another.	21 (11.1)	169 (88.9)	11 (6.5)	158 (93.5)
24- Assist the elderly person to transfer themselves from one place to another.	33 (17.4)	157 (82.6)	17 (10.8)	140 (89.2)
25- Employ the correct posture when transferring an elderly person from one place to another.	33 (17.4)	157 (82.6)	84 (53.5)	73 (46.5)
26- Provide support and/or materials necessary to position the elderly patient.	47 (24.7)	143 (75.3)	6 (4.2)	137 (95.8)
27- Assess the need to change the body position of the elderly person.	56 (29.5)	134 (70.5)	10 (7.5)	124 (92.5)
28- Employ the correct posture to position each part of the elderly person’s body correctly.	56 (29.5)	134 (70.5)	58 (43.3)	76 (56.7)
29- Change the body position of the elderly person when they are laying down.	90 (47.4)	100 (52.6)	16 (16)	84 (84)

^*^ NA= not applicable. ^†^proportions calculated on the number of caregivers who needed to perform the activity.

## Discussion

The results of this study show that care remains an activity performed predominantly by women, reinforcing information present in national and international studies regarding the role of women in care.(16-19) There is a need for special attention and encouragement for the self-care of women caregivers, since activities with the older person reduce their free time, bringing complications to their social life and even compromising their health.(20) The average age of informal caregivers was similar to others studies with caregivers of older people after stroke and dependent older people in general.(17,21) The demographic transition occurring in Brazil results in an increase in the older population, consequently, the number of older people caring for other older people increases.(22) Nevertheless, the support structures remain fragile and support networks are disorganized.(22)

Most caregivers were married, similar to other national studies.(17,19) Also, 38.4% of caregivers had jobs, differing from other national research.(17,19) Maintaining a healthy and active relationship with the spouse, reconciling employment and care activities becomes a difficult task. Some caregivers even abandon their jobs, putting their self-care in second place. With the precariousness of their relationships, many prioritize care activities for the older person.(23) The average education level of caregivers was similar to the national average;(1) however, it was lower than the average found in other studies developed in the same region.(21,24) Thus, nursing professionals play a fundamental role in finding strategies to educate informal caregivers according to their ability to understand, paying attention to their level of education.(2)

More than half of the responsibilities of caring for the older adult after a stroke were assumed by their children, which is similar to the findings of other studies with informal caregivers of older people after stroke and with caregivers of dependent older people in general.(18,19,21) Responsibility of children due to the duty to care for older parents is a subject discussed and investigated in recent years. In Brazilian culture, filial duty is defined as respect or devotion to parents, in which children feel the duty to repay the care dedicated by parents when they were children.(24) A study pointed out that the care provided by children, in most cases, is attributed to the fact that older people are unable to provide care for their spouses due to their disabilities.(21) The care of the older person after a stroke requires a long process of rehabilitation and effort from the caregiver and the care recipient.

In this study, most caregivers lived with the older person. This can be considered as a way to guarantee comprehensive care to patients with a functional disability after a stroke.(17) On the other hand, it requires great dedication. The median time of care for the older adult was five months, a period considered short to become able to care for a dependent older person after a stroke. Caregivers who provide care for a long time are more informed, as they learn from the daily care demands.(25) The median hours per week reported by caregivers as dedicated to caring for the older adult was 142.5 hours, above the median found in another study in the same region with relatives of dependent older people in general.(21) The high number of hours per week dedicated to the provision of care was because the caregiver reports being available full time to care for the older person, in tasks that include physical, emotional, and financial support. Such data are justified because the stroke is a disabling disease, with the caregiver having the function of providing care and/or managing care.(18)

More than half of the caregivers had some expense taken from their income, plus financial assistance from another person, due to the care of the older person after a stroke caused costs from spending on medication, adaptations at home, special diet, thickener, diapers, which is not always available in the public health system. Therefore, stroke generates costs both for the health system with hospitalization and rehabilitation and for the older person and their family at home.(23) Many caregivers need to withdraw part of their income to meet the basic care demands and providing a home for the whole family, often causing financial burden and impacts on the caregiver's quality of life.(18) Most older people had their income, around a minimum wage, showing low income as observed in another local study.(21)

Because stroke is a disabling disease, many older people return to their homes in need of different types of care. In the study, 68.4% of the older adults had moderate to severe disability, requiring the caregiver to perform more complex activities. Thus, the transition of care and hospital discharge planning must be carried out by nurses, aiming at guiding and preparing family caregivers to provide adequate conditions to meet the care demands of dependent older people, seeking to identify them and intervene according to the needs of each caregiver.

The hospitalization period of the older adults in this study had a median of 11 days, higher than the average length of stay due to stroke in Brazil, 7.5 days, from November 2016 to November 2017.(1) The median time of care for the older participants was 5 months. We believe that this period may have influenced the study's caregivers to acquire the ability to care since the average score of the caregivers in the *ECCIID-AVC* was 2.56 (± 0.42), varying from “demonstrates” (2) “demonstrates completely” (3). Although unknown, there is a time for learning to provide care, which involves the development of knowledge and skills, and psychological and relational aspects,(26) expressed in everyday life.

The caregiver often learns to deal with the care activities of a dependent older person through attempts and errors during day-to-day care at home.(27) However, this strategy can result in negative consequences for the care recipient. Each caregiver has also a demand for tasks according to the needs of the older adult. Thus, there is no established quantitative limit of activities they need to perform, but they must have the ability to perform them, exercise them safely and understand what they are doing. The informal caregiver should perform care while still in the hospital, supported by a multi-professional team, so that the caregiver learns and can perform these care activities adequately at home. This practice involves the planning of hospital discharge and the transition of care, from hospital to home, and the monitoring at home in a systematic way until it is assessed whether the caregiver has the autonomy to provide care.

The caregivers in this study showed to prepare the meal, paying attention to the recommended care at the time of hospital discharge regarding consistency (examples: pasty, mild, or normal) and the preparation of the diet. However, 36.9% of caregivers did not seek to stimulate motor skills on the side of the body of greater dependence on the older person during meals. This is a fundamental care for the rehabilitation of patients with hemiparesis after stroke and which should be reinforced in hospital discharge planning. (7) There was a low number of caregivers who performed feeding tube diets due to the few older people using it. Returning to the home with a feeding tube diet implies several changes in the life of the family, the individual, and the caregiver, requiring adequate guidance for those who will provide this care.(28) Only 15 of the 190 caregivers provided care for the older adult with a feeding tube diet and two of them had difficulty in performing this activity, ignoring basic care regarding the feeding tube diet, such as the use of water to unblock it. Likewise, findings from a Brazilian study report that obstruction of the tube is one of the main difficulties exposed by caregivers of dependent older people who return home.(29) Moreover, we highlight that activities which require knowledge and skills from the caregiver, such as handling the tube and preparing the diet, both orally and through the tube, are fundamental activities that must be addressed by the multi-professional team even when the older person is hospitalized. This action involves guidance and the observation of whether the caregiver can perform the activity with autonomy.

Most caregivers helped with the administration of medication according to the medical prescription, paying attention to the dose, times, route of administration, and storage, being a positive finding in the prevention of a new stroke and the recovery of the older person. A study with family caregivers of dependent older people identified that the care with medications represented one of the main activities with difficulties to be carried out at home.(27) Such difficulties refer to the lack of information and knowledge of the family members regarding their performance, especially regarding the identification and storage of medications, the expiration date, and changes in the prescribed doses.(27)

We found that, in the skin care activities, most caregivers performed the position rotation, hydrate the skin, and changed the diaper frequently. Patients with neurological disorders are more dependent on care, such as changing the position, hydrating the skin, nutritional assessment, and frequent intimate hygiene, and the lack of this care exposes the older person to a greater risk for the occurrence of pressure injuries.(7,30) The dressing was among the main activities that caregivers demonstrated the ability to perform. In the care of the older adult after a stroke, this activity requires skill, as it demands that caregivers pay attention to the use of comfortable clothing, start to wear it for the body member with the greatest dependence and, to undress, for the healthy body member.(7) Study-related to needs of family caregivers in caring for the dependent older person at home identified that caregivers lacked knowledge of how to perform the activity of dressing properly.(27) Although dressing is a less complex care activity, nurses must guide caregivers so that it is properly developed.

Regarding the activities of care with personal hygiene, eliminations, oral hygiene, and bed/shower bath, most of the caregivers were capable of carrying them out. These activities were facilitated by home adaptations that caregivers made and by having a bath chair, which facilitates the displacement of the older person. This finding differs from another study in which one of the main difficulties for family caregivers was the lack of equipment to provide home care.(27)

The transfer and positioning activities were performed by most of the caregivers in this study. However, most of them did not use adequate posture to perform the transfer and positioning, which is the main difficulty presented by caregivers. The inadequate posture of the caregiver to transfer the older person harms the health of the caregiver by performing the care with force and inadequate posture and also harms the older person. In this sense, we highlight the need for interventions performed by nursing professionals, together with the multidisciplinary team, to prevent musculoskeletal injuries in informal caregivers, which is associated with caring and promotes the well-being of both the caregiver and the older adult.(31)

As a stroke is a sudden disease, family members are often not prepared to care for a dependent older person, requiring the support of a formal health care network. In this perspective, the multi-professional teams of the primary health care units must provide training and support for these caregivers, especially in the initial phase of returning home. Since 2011, the Brazilian Ministry of Health has implemented a new modality of health care, substituting or complementing the existing ones, called Home Care, provided by primary health care (with support from the *Núcleo de Apoio à Saúde da Família* - NASF) or by home care services, according to the complexity of care. Home Care is characterized by a set of promotion, prevention, treatment, and rehabilitation actions provided at home, with guaranteed continuity of care.(32) Older people with more complex or even basic needs could be accompanied by this type of service in the home. However, the home care service must be improved and expanded so that all informal caregivers and the older adults after a stroke can count on this resource. It is necessary to increase the number of professionals in the primary health care teams so that it is possible to take care of the population's care demands.

The Brazilian Unified Health System and the Brazilian Unified Social Assistance System also need to implement other forms of formal care such as daycare centers. This would enable to reduce the burden on the caregiver's number of hours, as well as provide assistance to the older person according to their needs, reducing the risk of complications and hospital readmissions. For the most dependent older adults, home care can be an alternative to provide training and adequate support to informal caregivers in the provision of care at home or to enable institutionalization in cases of fragile families.

This investigation had limitations such as a non-probabilistic sample, containing only caregivers of older people from two specific hospitals in the south of Brazil, not allowing the results to be generalized to other regions of the country. Although the *ECCIID-AVC* was developed to be used in the outpatient and home context, we decided to apply it in this study, only in the outpatient context. Considering the country's socio-cultural diversity, we recommend new observational studies using *ECCIID-AVC* in other contexts and Brazilian regions. Another limitation found was the impossibility of comparison with other studies that used the same scale, since to date, there are no published studies that have used *ECCIID-AVC* in the national context.

This study contributes to the scientific knowledge of health professionals, especially nurses, as care managers and educators - both in hospital and outpatient settings, as well as at home - as they know what are the difficulties faced by informal caregivers. It also allows the elaboration of interventions aimed at teaching caregivers to develop these tasks in an appropriate way. This was the first study conducted in Brazil that used a specific scale for informal caregivers of older people after stroke with good reliability and validity (ECCIID-AVC), bringing results that encourage the planning of interventions for this population.

We conclude that most caregivers performed all essential care activities for dependent older people after stroke present in ECCIID-AVC, referring to oral feeding, medications, skin care, personal hygiene, bathing, eliminations, clothing, transfer, and positioning. However, most of the caregivers had difficulties in the transferring and positioning activities of the older adult due to the lack of guidance as to the proper posture to perform such activities.

Once again, we highlight the nurse's responsibility for identifying problems and developing interventions aimed at the needs of caregivers to direct them at all levels of health care. For an adequate transition of care, health professionals who work in different settings must assess and support the family and the older person in the demands of day-to-day care, paying attention to their needs. Consequently, there is an implication for the health system, regarding the need to expand social and health policies and programs that provide information and support appropriate to the needs of caregivers and the older person after stroke. Thus, health professionals should advocate for the expansion and articulation of the health care network, providing the necessary support to caregivers, both in planning discharge and in returning to the home of the dependent older person after a stroke.
